# Real-world treatment patterns in patients with dysmenorrhea in Japan: a retrospective database study

**DOI:** 10.3389/fgwh.2026.1775657

**Published:** 2026-06-24

**Authors:** Daisuke Goto, Masahiko Uchiyama, Amy Zhao, Yutaka Osuga

**Affiliations:** 1 Organon, Jersey City, NJ, United States; 2 Organon, Tokyo, Japan; 3 Teikyo Academic Research Center, Teikyo University, Tokyo, Japan; 4 Department of Obstetrics and Gynecology, Graduate School of Medicine, The University of Tokyo, Tokyo, Japan

**Keywords:** dysmenorrhea, estrogen progestin combination therapy, Japan, low-dose estrogen-progestin (LEP), menstrual cycle, prescription, real-world insurance claim review, treatment patterns

## Abstract

**Background:**

Dysmenorrhea poses significant disease burden beyond menstruation pain but patients are known to be undertreated.

**Objective:**

To describe treatment patterns after the diagnosis of dysmenorrhea using health insurance claims data from insured members in Japan.

**Methods:**

We analyzed treatment patterns following the diagnosis of dysmenorrhea in women aged 18–45 years using health insurance claims from multiple Japanese health insurance societies between January 1, 2017 and September 30, 2022. We examined the first-line and subsequent treatment lines up to the third line treatments.

**Results:**

A total of 70,981 patients were identified, with a mean age of 29.7 years old. The most common first-line treatments and their median [interquartile range] durations were as follows: low-dose estrogen–progestin (LEP; otherwise known as combined oral contraceptive) in 33,169 patients (46.7%) for 161 [65, 384] days, analgesics/hemostatic agents (symptomatic therapy) in 13,619 patients (19.2%) for 9 [5, 29] days, and Kampo medicine (Chinese traditional herbal medicine adapted to and evolved in Japan) in 10,835 patients (15.3%) for 62 [29, 134] days. Across the treatment sequences from the first to third lines, LEP was the most frequently prescribed therapy. The most frequent pattern was LEP plus analgesics/hemostatic agents (18,019; 25.4%), followed by LEP monotherapy (10,389;14.6%), which collectively accounted for approximately 40% of all treatments. Analgesics/hemostatic agents alone accounted for 8,523 (12.0%) and Kampo medicine alone for 4,806 (6.8%). The transition from analgesics/hemostatic agents to LEP occurred in 2,166 (3.1%) of patients.

**Conclusions:**

An analysis of treatment sequences up to the third line revealed that LEP was the most commonly prescribed medication for dysmenorrhea. The median duration of LEP use exceeded five months, suggesting that many patients continued LEP across multiple menstrual cycles and that LEP was well accepted and sustained as a treatment for dysmenorrhea in Japan.

## Introduction

Dysmenorrhea is a condition that occurs during menstruation, characterized mainly by lower abdominal and back pain, and may include bloating, nausea, headache, fatigue, loss of appetite, irritability, diarrhea, and depressive symptoms (1). The condition is primarily caused by endometrial inflammation and increased prostaglandin release (2). It adversely affects quality of life (3–5), often forcing affected women to change their daily plans due to menstrual pain (6). Although the prevalence of menstrual abnormalities among Japanese women is higher at >50%, the majority remain untreated (7, 8). Dysmenorrhea can be either primary or secondary; primary dysmenorrhea is characterized by menstrual pain driven by excessive prostaglandin-mediated uterine activity while lacking pelvic pathology (9). In contrast, secondary dysmenorrhea, which is accounted for approximately 10% of dysmenorrhea cases, stems from underlying pelvic pathology including but not limited to endometriosis (10).

According to the Japanese clinical guidelines, recommended treatments include low-dose estrogen–progestin combinations (LEP; otherwise known as combined oral contraceptive), progestin preparations, gonadotropin-releasing hormone analogs, Kampo medicine (traditional Japanese herbal medicine), antispasmodics, antifibrinolytics, and the levonorgestrel-releasing intrauterine system (11). Since prostaglandins produced in the endometrium play a central role in dysmenorrhea, nonsteroidal anti-inflammatory drugs, which inhibit prostaglandin synthesis, provide symptomatic relief (12).

LEP was approved for insurance coverage for dysmenorrhea in Japan in 2008, and since then, its use has steadily expanded for both primary and secondary dysmenorrhea (13–16). Previous studies demonstrated that in comparisons with cyclic administration, which induces withdrawal bleeding in a 28-day cycle, continuous LEP administration reduced the number of days with pain (17) and was more effective overall (18). Reflecting these findings, Canadian guidelines recommend continuous administration (19). Similarly, the Japanese guidelines recognize that continuous administration reduces the frequency of withdrawal bleedings, thereby reducing associated symptoms and menstrual pain significantly more than cyclic administration (16).

The last study that investigated treatment patterns for dysmenorrhea in Japan covered year 2009 through 2014 (13). In this previous study, LEP was prescribed for the first-line treatment in 37.7% of cases and Kampo medicine in 30.0%. Since 2014, newer LEP formulations, including generics, have become more widely available, and an increase in LEP prescriptions has been reported (14, 15). Given the availability of new LEP formulations and the passage of time since the previous study (13), the purpose of the current study is to provide an updated comprehensive picture of how dysmenorrhea is managed in the contemporary Japanese clinical practice so as to enable the clinical community identify the changes thus far in real-world dysmenorrhea treatment patterns and opportunities to further improve treatment decision-making. Therefore, we aimed to describe the contemporary treatment patterns among Japanese women diagnosed with dysmenorrhea; in particular, in light of the availability of new LEP formulations in the recent years in Japan, we hypothesized that we would observe the high LEP treatment share seen by Akiyama et al. (13) once again in this study. The study objective to fulfill the aforementioned aim was to describe the first three lines of treatment for dysmenorrhea and characterize the most common treatment patterns using one of the most comprehensive medical claims database in Japan.

## Methods

### Study design and data source

We used an anonymized health insurance claims database developed by JMDC Inc. (Tokyo, Japan; JMDC). The data were obtained via secondary-use permissions from health insurance societies. The database includes health insurance claims and demographic data, including the sex and age of insured individuals, diagnoses codes according to the International Classification of Diseases, 10th Revision, medical procedures, and prescription records. The database contained approximately 5 million insured individuals at the beginning of the study period in 2017, and around 10.44 million by the end of the period in 2022, covering roughly one-third of the 30 million individuals enrolled in Japan's social health insurance system.

### Study population

This was a retrospective study using a large Japanese health insurance claims database, the sample size was determined by the available population in the database rather than by a pre-specified power calculation. We included subscribers who met all of the following inclusion criteria and did not meet any exclusion criteria.

Inclusion criteria:

1. Individuals enrolled in a health insurance society and registered in the JMDC database.
2. Female patients aged between 18 and 45 years.
3. Those diagnosed with dysmenorrhea between January 1, 2017 and September 30, 2022. The definition of dysmenorrhea was patients whose International Classification of Diseases codes were N944, N945, or N946 and whose standard disease names in the Medical Information System Development Center were functional dysmenorrhea, menstrual pain, or dysmenorrhea.
4. Patients who had a second diagnosis of dysmenorrhea within three months after the initial diagnosis and who remained insured for at least one year following the initial diagnosis.
5. Patients who had been continuously insured for at least one year before the initial diagnosis of dysmenorrhea and had no prior diagnosis of dysmenorrhea during that period.

Exclusion criteria： Patients who met any of the following conditions during the study period (including before and after the diagnosis date) were excluded: those diagnosed with a hemorrhagic disorder or gynecologic cancer, and those who had undergone gynecologic surgery (hysterectomy, endometrial ablation, or myomectomy) or received hormonal therapy prior to the initial diagnosis of dysmenorrhea. The corresponding disease names and surgical procedures are listed in Supplementary Table 1.

### Data analysis

The primary items evaluated in this study were the treatment methods for dysmenorrhea and their prescription durations. The number and percentage of cases for each treatment category as well as the mean and median prescription durations were calculated for the first-line treatment of dysmenorrhea recorded in the medical claims. Furthermore, the first treatment change (second-line) and second treatment change (third-line) were analyzed to investigate how the first-line treatment was modified through to the third-line treatment. The number and percentage of cases for each treatment transition pattern were summarized.

**Figure 1:**
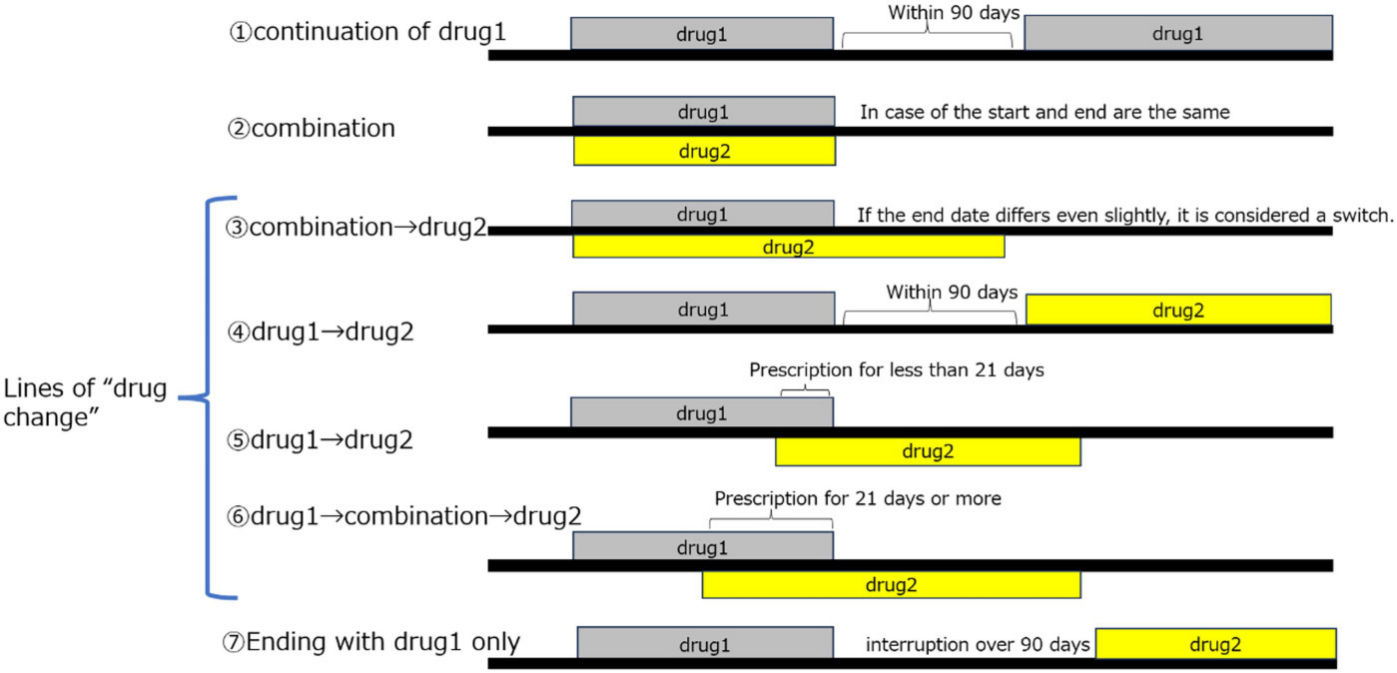
Identification of treatment line.

A treatment line was defined as a continuous prescription group of the same drug or combination of drugs. Each treatment line was characterized using the start and end dates, duration, and treatment category prescribed. If there was a gap of more than 90 days between two consecutive treatment lines, the treatment was considered to be discontinued. When there was an overlap of 21 days or more between consecutive treatment lines, the patient was considered to be receiving combination therapy (Figure 1). A treatment switch was defined as two consecutive treatment lines with less than 21 days of overlap and without a gap exceeding 90 days between them. Surgical procedures were treated as a single treatment line, and combination therapy including surgery was classified as a separate treatment category.

Treatment categories were defined as follows: (a) low-dose estrogen–progestin combinations (LEP; otherwise known as combined oral contraceptive), (b) medium/high-dose estrogen–progestin combinations, (c) progestins, (d) testosterone, (e) gonadotropin-releasing hormone analogs, (f) analgesics/hemostatic agents (symptomatic therapy), (g) Kampo medicine (Chinese traditional herbal medicine adapted to and evolved in Japan), (h) levonorgestrel intrauterine system, (i) surgery, and (j) combination therapy [two or more of categories [a]–[i]]. Details on each category are provided in Supplementary Table 2. These treatment-line identification methods follow those used in a preceding study (13), and in addition, this study was designed with further reference to previous real-world studies of dysmenorrhea (20) and other gynecological conditions (21, 22). Analgesic and hemostatic agents are both symptomatic treatments used for a short-period of time to address immediate clinical issues such as pain and menstrual bleeding and in this study, which aimed to have a focus on long-term treatments, symptomatic treatments were grouped into one category to avoid creating frequent changes to treatment lines and rendering it more complex to characterize long-term treatments. The overall treatment patterns from the first to third lines are summarized into seven sequence patterns based on chronological changes in treatment categories from the first line; when A, B, and C each represent a treatment category where “A” indicates the most frequently used treatment category starting from the first line, patterns are as follows: treatment with a single class of therapy A (“Single A”), treatment with a base therapy with additional class(es) of treatment (“A-based add-on B” and “A-based add-on Multiple”), transition from one to another (“A to B”), transition from one to another and back to the original (“A to B to A”), transition across three classes of treatment (“A to B to C”), and simultaneous use of two classes of therapies during the first line, first and second line, or all three lines (“A + B”) (detailed definitions are shown in Supplementary Table 3).

In addition, summary statistics were calculated for patients’ age, comorbidities, and Charleson Comorbidity Index (45) at the time of the dysmenorrhea diagnosis (see Supplementary Table 4 for the diagnosis codes). Our data analysis identified many patients with comorbid endometriosis and fibroids. As these comorbidities could affect the choice of treatment, we have conducted subgroup analyses by repeating the aforementioned analyses on patients who had endometriosis and then repeated the same for those with fibroids. These subgroup analyses were independently conducted.

Data were analyzed using SAS® version 9.4 or above (Cary, North Carolina, US; SAS Institute, Inc.). Continuous variables were expressed as the mean ± standard deviation or as the median [first quartile, third quartile]. Categorical variables were presented as frequencies (percentages).

### Human research ethics

The data used in this study was sourced from an existing anonymized insurance claims data database; our secondary use of an anonymized data set is considered as research that does not require review or approval by an institutional ethics committee according to the Ethical Guidelines for Medical and Health Research Involving Human Subjects in Japan (23).

## Results

A total of 70,981 patients were included in the analysis, with a mean age of 29.7 ± 7.8 years. Among them, 62,210 patients (87.6%) had dysmenorrhea only, while 8,771 (12.4%) also had menorrhagia. Endometriosis was present in 19,237 patients (27.1%), and uterine fibroids in 9,265 (13.1%). 42,138 patients (59.4%) had a Charleson Comorbidity Index score of zero (Table 1).

**Table 1:** Patient characteristics during 1 year baseline period.

Characteristics	*N*	%
Index year		
2017	5,410	7.6%
2018	8,075	11.4%
2019	11,870	16.7%
2020	15,689	22.1%
2021	18,479	26.0%
2022	11,458	16.1%
Mean (SD) age, years, on index date	29.70 (7.84)	
Age, years, category, *N* (%)		
18–19	6,901	9.7%
20–29	30,062	42.4%
30–39	23,950	33.7%
40–45	10,068	14.2%
Disease status, *N* (%)		
Dysmenorrhea only	62,210	87.6%
Both dysmenorrhea and HMB*	8,771	12.4%
Underlying conditions, *N* (%)		
Endometriosis	19,237	27.1%
Adenomyosis	3,448	4.9%
Fibrosis	9,265	13.1%
CCI category, *N* (%)		
0	42,138	59.4%
1	18,261	25.7%
2	6,145	8.7%
3	2,559	3.6%
4+	1,878	2.6%

*CCI, Charlson comorbidity index; HBM, heavy menstrual bleeding.

In the first-line treatment, low-dose estrogen–progestin combinations (LEP; otherwise known as combined oral contraceptive) alone was the most frequently prescribed (33,169 patients; 46.7%), followed by analgesics/hemostatic agents alone in 13,619 (19.2%) and Kampo medicine alone in 10,835 (15.3%) (Table 2). 4,310 patients (6.1%) received combination therapy including LEP as the first-line treatment, indicating that LEP was used in 37,479 (52.8%) of cases overall. The median [interquartile range] (mean ± standard deviation) durations of the main treatment categories were 161 [65, 384] (265 ± 285) days for LEP, 9 [5, 29] (34 ± 80) days for analgesics/hemostatic agents, and 62 [29, 134] (114 ± 154) days for Kampo medicine.

**Table 2:** Treatment patterns of the first treatment line.

First line treatment	*N*	%	Duration (Days)	
			Mean ± Std	Median [Q1, Q3]
LEP	33,169	46.7%	265 ± 285	161 [65, 384]
Analgesic/Hemostatic	13,619	19.2%	34 ± 80	9 [5, 29]
Kampo medicine	10,835	15.3%	114 ± 154	62 [29, 134]
Progestin	2,715	3.8%	165 ± 234	64 [14, 204]
LNG-IUS	1,510	2.1%	2 ± 5	2 [2, 2]
EPMedHigh	1,021	1.4%	35 ± 92	11 [11, 22]
GnRH	177	0.2%	88 ± 58	83 [38, 145]
Surgery	61	0.1%	1 ± 0	1 [1, 1]
Testosterone	2	0.0%	145 ± 154	145 [36, 254]
No Treatment	728	1.0%	NA	NA
Combination	7,144	10.1%	42 ± 87	11 [8, 38]
Combination - includes* LEP	4,310	6.1%		
Combination - includes* Analgesic/Hemostatic	5,650	8.0%		
Combination - includes* Kampo medicine	2,708	3.8%		
Combination - includes* Progestin	805	1.1%		
Combination - includes* LNG-IUS	278	0.4%		
Combination - includes* EPMedHigh	643	0.9%		
Combination - includes* GnRH	33	0.0%		
Combination - includes* Surgery	0	0.0%		
Combination - includes* Testosterone	0	0.0%		
Total	70,981			

EPMedHigh, medium/high dose estrogen–progestin; GnRH, gonadotropin-releasing hormone; Kampo medicine, Chinese traditional herbal medicine adapted to and evolved in Japan; LEP, low-dose estrogen–progestin (otherwise known as combined oral contraceptive); LNG-IUS, levonorgestrel intrauterine system; Std, standard deviation.

*Not mutually exclusive.

Across treatment sequences up to the third line, the most frequent treatment pattern involved the continuous use of LEP (Table 3). The most common pattern was the initial (first = line) LEP therapy followed by LEP therapy with an addition of analgesics/hemostatic agents (18,019; 25.4%); the second most common pattern was LEP monotherapy (10,389;14.6%); collectively, these accounted for approximately 40% of all treatment patterns. The third most common pattern was analgesics/hemostatic agents alone (8,523; 12.0%), and the fourth was Kampo medicine alone (4,806; 6.8%), with these four patterns together representing 58.8% of all treatments. Subsequent patterns were less frequent, with the next most common being initial Kampo medicine therapy followed by the addition of analgesics/hemostatic agents (2,963; 4.2%). Other notable pattern was a transition from analgesics/hemostatic agents to LEP, which occurred in 2,166 (3.1%) of cases. Progestin was prescribed for 2,715 patients (3.8%) as a first-line therapy. The pattern of using single progestin only continuously up to the third line accounted for 831 patients (1.2%). Full details on all treatment patterns are presented in Supplementary Table 5.

**Table 3:** Treatment patterns up to third line.

First line treatment	Treatment over initial three lines	*N*	Overall %	*N*	Overall %
LEP	All (any)	33,169	46.7%		
	LEP-based (add-on: Analgesic/Hemostatic)			18,019	25.4%
	LEP Only			10,389	14.6%
	LEP to Analgesic/Hemostatic			2,070	2.9%
	Other combinations			2,691	3.8%
Analgesic/Hemostatic	All (any)	13,619	19.2%		
	Analgesic/Hemostatic Only			8,523	12.0%
	Analgesic/Hemostatic to LEP			2,166	3.1%
	Analgesic/Hemostatic to Kampo medicine			742	1.0%
	Other combinations			2,188	3.1%
Kampo medicine	All (any)	10,835	15.3%		
	Kampo medicine Only			4,806	6.8%
	Kampo medicine-based (add-on: Analgesic/Hemostatic)			2,963	4.2%
	Kampo medicine to Analgesic/Hemostatic			1,694	2.4%
	Other combinations			1,372	1.9%
Combination	All (any)	7144	10.1%		
	LEP-based (add-on: Analgesic/Hemostatic)			2,174	3.1%
	LEP-based (add-on: Multiple)			1,088	1.5%
	Kampo medicine-based (add-on: Analgesic/Hemostatic)			1,006	1.4%
	Analgesic/Hemostatic-based (add-on: Kampo medicine)			318	0.4%
	Kampo medicine-based (add-on: Multiple)			219	0.3%
	Other combinations			2,339	3.3%
Progestin	All (any)	2715	3.8%		
	Progestin-based (add-on: Analgesic/Hemostatic)			936	1.3%
	Progestin Only			831	1.2%
	Other combinations			948	0.7%
LNG-IUS	All (any)	1510	2.1%		
	LNG-IUS Only			1,070	1.5%
	LNG-IUS to Analgesic/Hemostatic			376	0.5%
	Other combinations			64	0.1%
EPMedHigh	All (any)	1021	1.4%		
	EPMedHigh Only			294	0.4%
	EPMedHigh to LEP			197	0.3%
	Other combinations			530	1.1%
GnRH	All (any)	177	0.2%		
	GnRH to Progestin			28	0.0%
	GnRH Only			27	0.0%
	Other combinations			122	1.5%
Surgery	All (any)	61	0.1%		
	Surgery to Analgesic/Hemostatic			58	0.1%
	Surgery Only			3	0.0%
	Other combinations			0	0.0%
Testosterone	All (any)	2	0.0%		
	Testosterone Only			1	0.0%
	Testosterone to LEP			1	0.0%
	Other combinations			0	0.0%
No Treatment		728	1.0%		
Total		70,981			

EPMedHigh, medium/high dose estrogen–progestin; GnRH, gonadotropin-releasing hormone; Kampo medicine, Chinese traditional herbal medicine adapted to and evolved in Japan; LEP, low-dose estrogen–progestin (otherwise known as combined oral contraceptive); LNG-IUS, levonorgestrel intrauterine system.

Subgroup analyses for patients with endometriosis and fibrosis were conducted as these were frequently observed comorbidities (see Table 1) that might have affected treatment choices. The detailed results are presented in Supplementary Table 6. The mean age for the endometriosis subgroup was 30.06 ± 7.81 whereas the mean age for the fibrosis was 34.81 ± 7.33. The most frequent treatment pattern in a sequence up to the third line was the initial (first-line) LEP therapy followed by LEP therapy with an addition of analgesics/hemostatic agents (28.3% and 20.1%, respectively) for both endometriosis and fibrosis subgroups.

## Discussion

Among the first-line treatments for dysmenorrhea, low-dose estrogen–progestin combinations (LEP; otherwise known as combined oral contraceptive) monotherapy was the most frequent, being prescribed to nearly 5 out of 10 patients (Table 2). In the overall analysis of treatment sequences from the first to third lines (Table 3), the two most frequent treatment patterns of (LEP-based add-on Analgesic/Hemostatic: 25.4%) and (Single LEP: 14.6%) consistently included LEP throughout the treatment course. Collectively, these two patterns accounted for approximately 40% of all patients, indicating that LEP-based therapy has become well established as treatment for dysmenorrhea. While Japanese clinical guidelines recommend symptomatic therapy as the first-line treatment, followed by LEP, progestins, or a levonorgestrel intrauterine system when pain control is insufficient (11), our results suggest that LEP is frequently prescribed as a first-line treatment in the real-world clinical practices in Japan. In the present study, 27.1% of patients had endometriosis and 13.1% had uterine fibroids (Table 1). Previous studies demonstrated that LEP alleviated pain associated with endometriosis (24–26) and reduced menorrhagia related to uterine fibroids (27, 28).

The treatment choices for subgroups of patients with comorbid endometriosis and fibroids presented similar patterns. The most frequent treatment pattern remained the same for both subgroups (i.e., starting with mono-LEP therapy with added analgesic and/or hemostatic over the course of treatment). Our results show that the overall treatment strategy remains largely similar in these two subpopulations when compared to the entire population, indicating that treatment strategies do not substantially differ due to the presence of comorbid endometriosis and fibroids.

We hypothesized that the high share of LEP treatment reported by Akiyama et al. would again be observed. Our results were consistent with this hypothesis: LEP-based regimens accounted for a large proportion of first-line therapies and remained the most common pharmacological approach across treatment sequences, including both monotherapy and combination therapy with analgesic/hemostatic agents.

Including the next most common patterns, namely, those using only analgesics/hemostatic agents or only Kampo medicine, the four most frequent treatment patterns together accounted for approximately 6 in 10 cases (58.8%) whereas the remaining 41.2% received a number of treatment combinations. This result indicates that a wide range of therapeutic options are being utilized in clinical practice for dysmenorrhea management.

Prior study by Akiyama et al. (13), which also analyzed the first three lines of therapy for dysmenorrhea using data from 2009 through 2014, found that single-agent therapy accounted for a large majority; namely, 23.9% was with LEP alone, 10.4% with analgesics/hemostatic agents alone, and 17.7% with Kampo medicine alone, totaling 52.0%. Although detailed information on combination therapy was not available in that study, the patients who initially received LEP and were later switched to LEP combined with other therapies represented 4.1% of the total study population, and when this was included, the total percentage reached 56.1% (13). When we compare the findings of Akiyama et al. (13) with our analysis that used more recent data, the pattern of first-line LEP followed by the addition of analgesics/hemostatic agents was notably prevalent, accounting for 25.4% overall. While the use of analgesics/hemostatic agents monotherapy remained almost unchanged (+1.6%), Kampo medicine monotherapy decreased by 10.9% and LEP monotherapy decreased by approximately 9.3%. The finding shows that LEP supplemented with analgesics/hemostatic agents for symptomatic relief has become an important therapeutic strategy. In addition, compared to a report by Akiyama et al. where the rate of progestin use in first-line treatment was 1.0% (13), the rate was higher by 2.8 percentage points in this survey. This also gives a glimpse into the increasing number of aggressive treatments.

The uniqueness of the LEP therapy is its extended duration of use, the median duration of LEP use in first-line treatment exceeded five months (161 days), which was much longer than that of analgesics/hemostatic agents or Kampo medicine (Table 2). The long duration of LEP use is reflective of the characteristics of the drug, and many patients who continued LEP therapy may have experienced multiple menstrual cycles and may otherwise have caused dysmenorrheic pain. This finding potentially implies patients’ acceptance of LEP treatment. In a Japanese interview survey, all participants who had continued oral therapy for more than six months (*N* = 10) cited satisfaction with being able to manage menstruation through the use of medication as the primary reason for continuation, and 8 of the 10 also reported that their symptoms had lessened and that their daily activities were no longer affected (29). Another study reported that the factor with the strongest impact on the choice of treatment for dysmenorrhea was the “method of administration”, followed by the “possibility of irregular bleeding” (30). These attributes of LEP therapy may have contributed to long-term adherence.

The median duration of first-line LEP use (161 days) in our analysis was similar to the findings by Akiyama et al. (13) (206 days for primary dysmenorrhea and 174 days for secondary dysmenorrhea) (Table 2). Furthermore, the median duration of Kampo treatment in the present study was 62 days, which is similar to the findings by Akiyama et al. (13). (69 days for primary and 70 days for secondary dysmenorrhea). The present results indicate that the duration of first-line treatment by drug type has remained largely unchanged. The prevalence of endometriosis, uterine fibroids, and adenomyosis has also remained largely unchanged compared with the findings of Akiyama et al. (13).

The number of cases analyzed increased from 6,315 in Akiyama et al.'s study (1,263 patients per year on average) (13) to 70,981 in the present study (14,196 patients per year on average), an approximately 11-fold increase. Since 2014, the annual number of insured individuals in the JMDC database has tripled (13, 31); similarly, the likelihood of receiving medical consultation for dysmenorrhea in Japan has increased since 2014, with follow-up visits showing a particularly notable rise, increasing approximately 2.5-fold between 2014 and 2021 (12). This change likely reflects greater awareness of menstruation-related conditions (32, 33), changes in health-seeking behavior (33), reduced social stigma (14, 34), and better access to healthcare services (33) rather than an increase in the prevalence of the disease. Together with the higher percentage of patients using LEP and the longer treatment duration observed in the present study, may partly explain changes in patient awareness as outlined above. The treatment patterns observed in this study, especially continued prioritization of LEP-based therapies seen in both our study and Akiyama et al. (13), may be reflective of pursuit to effective treatments for dysmenorrhea, which carries significant psychological burden associated with recurrent and anticipated menstrual pain. Contrasting to the preference toward LEPs, the sustained share of Kampo medication seen in the aforementioned studies, while declined between the studies, demonstrate preference toward herbal medicine by a sizable share of patients.

Despite the trend, the existing literature still suggests that there still are patients who do not seek medical care, discounting the need to address the condition; this hesitation is also seen in patients’ hesitation to seek sick leaves (35–40). In the meantime, there has been a report that online awareness campaigns effectively encourage individuals who experience menstrual issues, particularly those who remain undiagnosed or have not received care, to seek care (41). Expanding public recognition of dysmenorrhea as a condition with effective pharmacological interventions and encouraging appropriate medical consultations to affected patients may further increase access to effective treatments, such as LEP and reduce the number of women who suffer from dysmenorrhea.

Although our analysis focused on pharmacological therapies captured in insurance claims data, emerging evidence indicates that structured exercise programs and other physical therapies may alleviate menstrual pain, improve physical function, and reduce reliance on analgesic medications, suggesting a potential role for these interventions as either adjuncts or alternatives to pharmacotherapy (42–44). Future studies should therefore explore combined pharmacological and non-pharmacological approaches to support more comprehensive management of dysmenorrhea.

## Limitations

The severity of dysmenorrhea symptoms in this study population was unknown, and a shorter treatment duration does not necessarily indicate inadequate therapy. The database used in this study consisted only of data from selected health insurance societies that had granted permission for secondary use, and non-participating insurers were excluded. Over-the-counter medications were not included in the analysis. Patients who successfully managed their symptoms with over-the-counter medications may have been less likely to seek a follow-up visit, leading to selection bias toward patients with more persistent or severe symptoms. In Japan, hormonal agents for dysmenorrhea are not available as over-the-counter medications, and over-the-counter medications was unlikely to affect the initiation or sequencing of prescription hormonal therapies observed in this database. Also, the requirement for a second diagnosis of dysmenorrhea within three months (inclusion criterion 4) may have excluded women who sought medical care only once and subsequently self-managed their symptoms including those who used over-the-counter analgesics. Patients were not followed across different employers. Therefore, individuals who changed employment might have appeared more than once, or partially or fully excluded from the analysis depending on how frequently they changed their employment.

## Conclusion

Low-dose estrogen–progestin combinations (LEP; otherwise known as combined oral contraceptive) was the most commonly prescribed treatment for dysmenorrhea. Our hypothesis was that the high share of LEP treatment previously reported in Japanese claims data would persist over time and it was supported, as LEP-based regimens remained the most common pharmacological approach for dysmenorrhea in our newer cohort. Across treatment sequences up to the third line, 25.4% of patients received first-line LEP followed by the addition of analgesics/hemostatic agents, and 14.6% received LEP monotherapy. Collectively, these two patterns accounted for 40% of all treatment regimens. The median duration of LEP monotherapy as the first-line treatment exceeded five months, which was longer than that of analgesics/hemostatic agents or Kampo medicine, suggesting that patients who initiated LEP therapy were able to continue treatment for a longer period. Many patients who continued LEP likely experienced multiple menstrual cycles that may have otherwise caused dysmenorrheic pain, indicating that LEP was well accepted among dysmenorrhea patients in Japan.

## Data Availability

The data analyzed in this study is subject to the following licenses/restrictions: The data that support the findings of this study are available from JMDC Inc.; however, these were used under the license for the current study and are therefore not publicly available. Requests to access these datasets should be directed to the corresponding author, Daisuke Goto at daisuke.goto1@organon.com.
