# Supplementary material for: Real-world treatment patterns in patients with dysmenorrhea in Japan: a retrospective database study
Source: Front Glob Womens Health. 2026 Jun 24;7:1775657. doi: 10.3389/fgwh.2026.1775657 (PMC13341935; doi:10.3389/fgwh.2026.1775657)
Supplement: Supplementary file 3 [file Table3.docx]

Supplementary Table 3. Defining Treatment Patterns

| Treatment Pattern | 1st. Line | 2nd. Line | 3rd. Line |
| --- | --- | --- | --- |
| Single A | A | A | A |
|  | A | A | No |
|  | A | No | No |
| A-Based add-on B | A | A+B | A |
|  | A | A | A+B |
|  | A+B | A+B | A |
|  | A+B | A | A+B |
|  | A | A+B | A+B |
|  | A+B | A | A |
|  | A+B | A | No |
|  | A | A+B | No |
| A-Based add-on Multiple* |  |  |  |
| A to B | A | A | B |
|  | A | B | B |
|  | A | B | No |
|  | A+B | A | B |
|  | A | A+B | B |
|  | A | B | A+B |
| A to B to A | A | B | A |
| A to B to C | A | B | C |
|  | A | B | B+C |
|  | A+B | B | C |
|  | A | B+C | C |
|  | A+B | B+C | C |
|  | A | A+B | B+C |
|  | A | A+B | C |
| A + B | A +B | No | No |
|  | A +B | A+B | No |
|  | A +B | A+B | A+B |

*A is present in all lines and more than one treatment was added throughout the lines.
