# Supplementary material for: Real-world treatment patterns in patients with dysmenorrhea in Japan: a retrospective database study
Source: Front Glob Womens Health. 2026 Jun 24;7:1775657. doi: 10.3389/fgwh.2026.1775657 (PMC13341935; doi:10.3389/fgwh.2026.1775657)
Supplement: Supplementary file 6 [file Table6.docx]

**Supplementary Table 6**

Supplementary Table 6-1a. Patient Characteristics During 1 Year Baseline Period for Patients with Endometriosis

| **Characteristics** | **N** | **%** |
| --- | --- | --- |
| **Index year** |  |  |
| 2017 | 1,458 | 7.6% |
| 2018 | 2,127 | 11.1% |
| 2019 | 2,969 | 15.4% |
| 2020 | 4,221 | 21.9% |
| 2021 | 5,227 | 27.2% |
| 2022 | 3,235 | 16.8% |
| **Mean (SD) age, years, on index date** | 30.06 (7.81) |  |
| **Age, years, category, N (%)** |  |  |
| 18-19 | 1,624 | 8.4% |
| 20-29 | 8,065 | 41.9% |
| 30-39 | 6,646 | 34.5% |
| 40-45 | 2,902 | 15.1% |
| **Disease status, N (%)** |  |  |
| Dysmenorrhea only | 16,753 | 87.1% |
| Both dysmenorrhea and HMB* | 2,484 | 12.9% |
| **Underlying conditions, N (%)** |  |  |
| Endometriosis | 19,237 | 100.0% |
| Adenomyosis | 3,448 | 17.9% |
| Fibrosis | 2,270 | 11.8% |
| **CCI category, N (%)** |  |  |
| 0 | 11,400 | 59.3% |
| 1 | 5,008 | 26.0% |
| 2 | 1,628 | 8.5% |
| 3 | 705 | 3.7% |
| 4+ | 496 | 2.6% |

**Abbreviations: CCI, Charlson Comorbidity Index; HBM, heavy menstrual bleeding*

Supplementary Table 6-1b. Patient Characteristics During 1 Year Baseline Period for Patients with Fibrosis

| **Characteristics** | **N** | **%** |
| --- | --- | --- |
| **Index year** |  |  |
| 2017 | 738 | 8.0% |
| 2018 | 1,126 | 12.2% |
| 2019 | 1,493 | 16.1% |
| 2020 | 1,918 | 20.7% |
| 2021 | 2,448 | 26.4% |
| 2022 | 1,542 | 16.6% |
| **Mean (SD) age, years, on index date** | 34.81 (7.33) |  |
| **Age, years, category, N (%)** |  |  |
| 18-19 | 264 | 2.8% |
| 20-29 | 2,006 | 21.7% |
| 30-39 | 3,981 | 43.0% |
| 40-45 | 3,014 | 32.5% |
| **Disease status, N (%)** |  |  |
| Dysmenorrhea only | 7,528 | 81.3% |
| Both dysmenorrhea and HMB* | 1,737 | 18.7% |
| **Underlying conditions, N (%)** |  |  |
| Endometriosis | 6,995 | 75.5% |
| Adenomyosis | 8,745 | 94.4% |
| Fibrosis | 9,265 | 100.0% |
| **CCI category, N (%)** |  |  |
| 0 | 5,015 | 54.1% |
| 1 | 2,413 | 26.0% |
| 2 | 1,016 | 11.0% |
| 3 | 446 | 4.8% |
| 4+ | 375 | 4.0% |

**Abbreviations: CCI, Charlson Comorbidity Index; HBM, heavy menstrual bleeding*

Supplementary Table 6-2a. Treatment Patterns of the First Treatment Line for Patients with Endometriosis

|  | **N** | **%** | **Duration** | |
| --- | --- | --- | --- | --- |
|  |  |  | **Mean ± Std** | **Median [Q1, Q3]** |
| LEP | 9,920 | 51.6% | 266 ± 287 | 161 [64, 385] |
| Analgesic/Hemostatic | 3,592 | 18.7% | 26 ± 54 | 8 [5, 21] |
| Kampo medicine | 1,322 | 6.9% | 215 ± 265 | 108 [31, 295] |
| Progestin | 1,234 | 6.4% | 94 ± 145 | 43 [17, 106] |
| LNG-IUS | 490 | 2.5% | 3 ± 7 | 2 [2, 2] |
| EPMedHigh | 194 | 1.0% | 26 ± 48 | 11 [11, 16] |
| GnRH | 154 | 0.8% | 0 ± 0 | 0 [0, 0] |
| Surgery | 113 | 0.6% | 87 ± 56 | 80 [42, 143] |
| Testosterone | 15 | 0.1% | 1 ± 0 | 1 [1, 1] |
| No Treatment | 2 | 0.0% | 145 ± 154 | 145 [36, 254] |
| Combination | 2,201 | 11.4% | 40 ± 91 | 11 [8, 36] |
| Combination - includes* LEP | 1,421 | 7.4% |  |  |
| Combination - includes* Analgesic/Hemostatic | 1,881 | 9.8% |  |  |
| Combination - includes* Kampo medicine | 574 | 3.0% |  |  |
| Combination - includes* Progestin | 330 | 1.7% |  |  |
| Combination - includes* LNG-IUS | 113 | 0.6% |  |  |
| Combination - includes* EPMedHigh | 121 | 0.6% |  |  |
| Combination - includes* GnRH | 16 | 0.1% |  |  |
| Combination - includes* Surgery | 16 | 0.1% |  |  |
| Combination - includes* Testosterone | 0 | 0.0% |  |  |
| **Total** | **19,237** |  |  |  |

* Not mutually exclusive

EPMedHigh: medium/high dose estrogen–progestin; GnRH :Gonadotropin-Releasing Hormone; Kampo medicine: Chinese traditional herbal medicine adapted to and evolved in Japan; LEP: low-dose estrogen–progestin(otherwise known as combined oral contraceptive); LNG-IUS: levonorgestrel intrauterine system; Std: standard deviation.

Supplementary Table 6-2b. Treatment Patterns of the First Treatment Line for Patients with Fibrosis

|  | **N** | **%** | **Duration** | |
| --- | --- | --- | --- | --- |
|  |  |  | **Mean ± Std** | **Median [Q1, Q3]** |
| LEP | 3,501 | 37.8% | 261 ± 291 | 150 [58, 375] |
| Analgesic/Hemostatic | 2,114 | 22.8% | 34 ± 77 | 11 [5, 31] |
| Kampo medicine | 1,362 | 14.7% | 107 ± 147 | 57 [26, 125] |
| Progestin | 573 | 6.2% | 215 ± 269 | 112 [29, 294] |
| LNG-IUS | 378 | 4.1% | 3 ± 6 | 2 [2, 2] |
| EPMedHigh | 162 | 1.7% | 30 ± 68 | 13 [11, 22] |
| GnRH | 110 | 1.2% | 90 ± 58 | 81 [40, 146] |
| No Treatment | 86 | 0.9% | 0 ± 0 | 0 [0, 0] |
| Surgery | 51 | 0.6% | 1 ± 0 | 1 [1, 1] |
| Testosterone | 0 | 0.0% | NA | NA |
| Combination | 928 | 10.0% | 43 ± 86 | 15 [8, 38] |
| Combination - includes* LEP | 438 | 4.7% |  |  |
| Combination - includes* Analgesic/Hemostatic | 781 | 8.4% |  |  |
| Combination - includes* Kampo medicine | 405 | 4.4% |  |  |
| Combination - includes* Progestin | 108 | 1.2% |  |  |
| Combination - includes* LNG-IUS | 69 | 0.7% |  |  |
| Combination - includes* EPMedHigh | 64 | 0.7% |  |  |
| Combination - includes* GnRH | 19 | 0.2% |  |  |
| Combination - includes* Surgery | 19 | 0.2% |  |  |
| Combination - includes* Testosterone | 0 | 0.0% |  |  |
| **Total** | **9,265** |  |  |  |

* Not mutually exclusive

EPMedHigh: medium/high dose estrogen–progestin; GnRH :Gonadotropin-Releasing Hormone; Kampo medicine: Chinese traditional herbal medicine adapted to and evolved in Japan; LEP: low-dose estrogen–progestin(otherwise known as combined oral contraceptive); LNG-IUS: levonorgestrel intrauterine system; Std: standard deviation.

Supplementary Table 6-3a. Treatment patterns up to third line for Patients with Endometriosis

| **1st line** | **Treatment over initial 3 lines** | **N** | **Overall %** | **N** | **Overall %** |
| --- | --- | --- | --- | --- | --- |
| LEP | All(any) | 9,920 | 51.6% |  |  |
|  | LEP-based add-on Analgesic/Hemostatic |  |  | 5,444 | 28.3% |
|  | Single LEP |  |  | 3,002 | 15.6% |
|  | LEP to Analgesic/Hemostatic |  |  | 633 | 3.3% |
|  | Other combinations |  |  | 841 | 4.4% |
| Analgesic/Hemostatic | All(any) | 3,592 | 18.7% |  |  |
|  | Single Analgesic/Hemostatic |  |  | 1,985 | 10.3% |
|  | Analgesic/Hemostatic to LEP |  |  | 740 | 3.8% |
|  | Analgesic/Hemostatic to Progestin |  |  | 180 | 0.9% |
|  | Other combinations |  |  | 687 | 3.6% |
| Kampo medicine | All(any) | 1,234 | 6.4% |  |  |
|  | Single Kampo medicine |  |  | 457 | 2.4% |
|  | Kampo medicine-based add-on Analgesic/Hemostatic |  |  | 306 | 1.6% |
|  | Kampo medicine to Analgesic/Hemostatic |  |  | 166 | 0.9% |
|  | Other combinations |  |  | 305 | 1.6% |
| Combination | All(any) | 2,201 | 11.4% |  |  |
|  | LEP-based add-on Analgesic/Hemostatic |  |  | 533 | 2.8% |
|  | Analgesic/Hemostatic to LEP |  |  | 307 | 1.6% |
|  | LEP-based add-on Multiple |  |  | 297 | 1.5% |
|  | Other combinations |  |  | 1,064 | 5.5% |
| Progestin | All(any) | 1,322 | 6.9% |  |  |
|  | Progestin-based add-on Analgesic/Hemostatic |  |  | 604 | 3.1% |
|  | Single Progestin |  |  | 387 | 2.0% |
|  | Progestin to Analgesic/Hemostatic |  |  | 93 | 0.5% |
|  | Other combinations |  |  | 238 | 1.2% |
| LNG-IUS | All(any) | 490 | 2.5% |  |  |
|  | Single LNG-IUS |  |  | 339 | 1.8% |
|  | LNG-IUS to Analgesic/Hemostatic |  |  | 127 | 0.7% |
|  | LNG-IUS to Kampo medicine |  |  | 6 | 0.0% |
|  | Other combinations |  |  | 18 | 0.1% |
| EPMedHigh | All(any) | 194 | 1.0% |  |  |
|  | Single EPMedHigh |  |  | 50 | 0.3% |
|  | EPMedHigh to LEP |  |  | 45 | 0.2% |
|  | EPMedHigh to LEP to Analgesic/Hemostatic |  |  | 38 | 0.2% |
|  | Other combinations |  |  | 61 | 0.3% |
| GnRH | All(any) | 113 | 0.6% |  |  |
|  | GnRH to Progestin |  |  | 22 | 0.1% |
|  | GnRH to Progestin to Analgesic/Hemostatic |  |  | 16 | 0.1% |
|  | GnRH-based add-on Analgesic/Hemostatic |  |  | 15 | 0.1% |
|  | Other combinations |  |  | 60 | 0.3% |
| Surgery | All(any) | 15 | 0.1% |  |  |
|  | Surgery to Analgesic/Hemostatic |  |  | 14 | 0.1% |
|  | Single Surgery |  |  | 1 | 0.0% |
|  | Other combinations |  |  | 0 | 0.0% |
| Testosterone | All(any) | 2 | 0.0% |  |  |
|  | Single Testosterone |  |  | 1 | 0.0% |
|  | Testosterone to LEP |  |  | 1 | 0.0% |
|  | Other combinations |  |  | 0 | 0.0% |
| No Treatment |  | 154 |  | 154 |  |
| **Total** |  | **19,237** |  |  |  |

EPMedHigh: medium/high dose estrogen–progestin; GnRH:Gonadotropin-Releasing Hormone; Kampo medicine: Chinese traditional herbal medicine adapted to and evolved in Japan; LEP: low-dose estrogen–progestin (otherwise known as combined oral contraceptive); LNG-IUS: levonorgestrel intrauterine system.

Supplementary Table 6-3b. Treatment patterns up to third line for Patients with Fibrosis

| **1st line** | **Treatment over initial 3 lines** | **N** | **Overall %** | **N** | **Overall %** |
| --- | --- | --- | --- | --- | --- |
| LEP | All(any) | 3,501 | 37.8% |  |  |
|  | LEP-based add-on Analgesic/Hemostatic |  |  | 1,858 | 20.1% |
|  | Single LEP |  |  | 1,017 | 11.0% |
|  | LEP to Analgesic/Hemostatic |  |  | 259 | 2.8% |
|  | Other combinations |  |  | 367 | 4.0% |
| Analgesic/Hemostatic | All(any) | 2,114 | 22.8% |  |  |
|  | Single Analgesic/Hemostatic |  |  | 1,289 | 13.9% |
|  | Analgesic/Hemostatic to LEP |  |  | 269 | 2.9% |
|  | Analgesic/Hemostatic to Kampo medicine |  |  | 116 | 1.3% |
|  | Other combinations |  |  | 440 | 4.7% |
| Kampo medicine | All(any) | 1,362 | 14.7% |  |  |
|  | Single Kampo medicine |  |  | 498 | 5.4% |
|  | Kampo medicine-based add-on Analgesic/Hemostatic |  |  | 396 | 4.3% |
|  | Kampo medicine to Analgesic/Hemostatic |  |  | 226 | 2.4% |
|  | Other combinations |  |  | 242 | 2.6% |
| Combination | All(any) | 928 | 10.0% |  |  |
|  | LEP-based add-on Analgesic/Hemostatic |  |  | 150 | 1.6% |
|  | LEP-based add-on Multiple |  |  | 112 | 1.2% |
|  | Analgesic/Hemostatic to Kampo medicine |  |  | 88 | 0.9% |
|  | Other combinations |  |  | 578 | 6.2% |
| Progestin | All(any) | 573 | 6.2% |  |  |
|  | Progestin-based add-on Analgesic/Hemostatic |  |  | 246 | 2.7% |
|  | Single Progestin |  |  | 169 | 1.8% |
|  | Progestin to Analgesic/Hemostatic |  |  | 53 | 0.6% |
|  | Other combinations |  |  | 105 | 1.1% |
| LNG-IUS | All(any) | 378 | 4.1% |  |  |
|  | Single LNG-IUS |  |  | 253 | 2.7% |
|  | LNG-IUS to Analgesic/Hemostatic |  |  | 101 | 1.1% |
|  | LNG-IUS to Kampo medicine |  |  | 6 | 0.1% |
|  | Other combinations |  |  | 18 | 0.2% |
| EPMedHigh | All(any) | 162 | 1.7% |  |  |
|  | Single EPMedHigh |  |  | 48 | 0.5% |
|  | EPMedHigh to LEP |  |  | 25 | 0.3% |
|  | EPMedHigh to Analgesic/Hemostatic |  |  | 23 | 0.2% |
|  | Other combinations |  |  | 66 | 0.7% |
| GnRH | All(any) | 110 | 1.2% |  |  |
|  | Single GnRH |  |  | 18 | 0.2% |
|  | GnRH to Progestin |  |  | 16 | 0.2% |
|  | GnRH to Progestin to Analgesic/Hemostatic |  |  | 12 | 0.1% |
|  | Other combinations |  |  | 64 | 0.7% |
| Surgery | All(any) | 51 | 0.6% |  |  |
|  | Surgery to Analgesic/Hemostatic |  |  | 49 | 0.5% |
|  | Single Surgery |  |  | 2 | 0.0% |
|  | Other combinations |  |  | 0 | 0.0% |
| Testosterone | All(any) | 0 | 0.0% |  |  |
|  | Other combinations |  |  | 0 | 0.0% |
| No Treatment |  | 86 | 0.9% | 86 | 0.9% |
| **Total** |  | **9,265** |  |  |  |

EPMedHigh: medium/high dose estrogen–progestin; GnRH:Gonadotropin-Releasing Hormone; Kampo medicine: Chinese traditional herbal medicine adapted to and evolved in Japan; LEP: low-dose estrogen–progestin (otherwise known as combined oral contraceptive); LNG-IUS: levonorgestrel intrauterine system.
